# The association of latent toxoplasmosis and level of serum testosterone in humans

**DOI:** 10.1186/s13104-018-3468-5

**Published:** 2018-06-08

**Authors:** Nima Zouei, Saeedeh Shojaee, Mehdi Mohebali, Hossein Keshavarz

**Affiliations:** Department of Medical Parasitology and Mycology, Tehran University of Medical Sciences, Pour Sina St., Ghods St., Enghelab St., Tehran, 1417613191 Iran

**Keywords:** *Toxoplasma gondii*, Testosterone, Electro chemiluminescence immunoassay, Latent toxoplasmosis

## Abstract

**Objectives:**

Latent toxoplasmosis modifies various hormones and behaviors in infected hosts and possibly involves in etiology of different neurologic and psychiatric disorders. The aim of the current study was to assess possible associations between latent toxoplasmosis and testosterone concentration in *Toxoplasma* infected and free subjects. Briefly, 18–49 year-old participated in the study. After collected blood samples, sera were analyzed for the detection of anti-*Toxoplasma* IgG antibody. Totally, 76 positive sera were selected as study group (38 from men and 38 from women) and a same number of negative sera as control group.

**Results:**

Comparison of testosterone concentrations and control groups showed that testosterone concentration in study group was higher than that in control group with statistically significant difference (P = 0.024 and P = 0.043 for men and women, respectively). Significant differences were found in testosterone concentrations and anti-*Toxoplasma* IgG antibody levels in study and control groups (P < 0.05). Toxoplasmosis can affect the mean concentration of serum testosterone in human. Alteration of testosterone during latent toxoplasmosis can result in alterations in behavioral, physiologic and immunological parameters in long time.

## Introduction

*Toxoplasma gondii* is one of the most common parasitic protozoans in humans which cause toxoplasmosis [1]. The prevalence of toxoplasmosis varies from 20 to 80% in different parts of the world. Humans become infected through the oral route by the consumption of raw or undercooked meat contaminated with tissue cysts and other food products, water or vegetables contaminated with oocysts. Congenital infection can occur via vertical transmission of *T. gondii* tachyzoites from pregnant mother to developing fetus during the primary infection that could be life threatening for the fetus [2]. Therefore, the accurate diagnosis of acute maternal toxoplasmosis in pregnant women is critical [3]. Latent toxoplasmosis is clinically asymptomatic in immunocompetent hosts. However, the infection is usually long-lasting characterized by the presence of *Toxoplasma* cysts, typically in nervous and muscular tissues. Furthermore, the infection mostly results in a lifetime protective immunity (humoral and cellular) to reinfection, presenting low levels of anti-*Toxoplasma* IgG in serum of infected individuals [4]. Latent toxoplasmosis is known to induce various hormonal and behavioral changes in infected humans and animals and may be involved in etiology of different neurologic and psychiatric disorders [5–7]. Infected mice and rats have been shown to suffer from impaired motor neuron performance and coordination, deficit learning and reduced avoidance of open spaces and predators [8–12]. These are believed to be evolutionary mechanisms to increase the chance of hosts being eaten by felines [13]. Furthermore, latent toxoplasmosis increases chance of giving birth to males in humans and mice [14, 15]. Patients with changed testosterone levels may experience physical symptoms such as dermal hyper reactions including irritation, erythema, hirsutism and acne as well as abnormal growth of muscles, kidney failure and psychological deficits such as mood swings, depression and anxiety [16]. An effect of latent toxoplasmosis on serum testosterone changes is still being discussed by researchers. Published data have shown increased and decreased testosterone levels associated with *T. gondii* seropositivity in humans [17–19]. In the current study, effects of latent toxoplasmosis on serum testosterone were assessed in men and women.

## Main text

### Methods

#### Samples and patients

In this case–control study, 18–49 year-old men and women with no clinical complications were participated. Blood samples were collected in clinical laboratories in Tehran, May–September 2013. Information sheets were prepared and demographic questionnaires completed for the participants. Then, 3 ml of whole blood were collected and sera were tested for the detection of anti-*Toxoplasma* IgG antibody. In total, 76 positive sera were selected as study group (equally from men and women) and further 76 negative as control group.

#### Serological tests

Enzyme-linked immunosorbent assay (ELISA) was used to detection of anti-*Toxoplasma* IgG antibody in blood sera. The cut off values of optical densities (OD) were calculated according to a protocol by Hillyer et al. [20]. The OD of each sample was compared with cut off and recorded.

#### Antigen preparation

Antigen was prepared as previously described [21]. The RH strain of *T. gondii* was obtained from the Department of Parasitology, Tehran University of Medical Sciences, Tehran, Iran. Briefly, tachyzoites of *T. gondii,* RH strain were inoculated intraperitoneally into BALB/c mice. After 48–72 h, tachyzoites were collected using peritoneum washing with sterile normal saline (pH 7.2). Tachyzoites were washed with phosphate-buffered saline (PBS pH 7.4) for three times, sonicated in PBS (pH 7.4) and centrifuged at 12,000 g for 1 h at 4 °C. Then, supernatants were collected and protein density was assessed using Bradford method. Animal experiments were done according to Committee for the Update of the Guide for the Care and Use of Laboratory Animals and approved by the Ethical Committee of Tehran University of Medical Sciences for the use of laboratory animals.

#### Detection of anti-Toxoplasma IgG antibody using ELISA technique

The 96-well microplates (Nunk, Germany) were coated with 5 µg/ml of soluble antigen of *T. gondii* RH strain in carbonate-bicarbonate buffer (pH 9.6) and stored at 4 °C. Plates were washed for three times with PBST (PBS, 0.05% tween 20) and sera were diluted 1:200 in PBST and 100 µl from diluted sera was added to each well of microplate. After incubation for 1 h at 37 °C and three times of washing, 100 µl of anti-human IgG conjugated with hourseradish peroxidase (HRP) (Dako, Denmark) diluted 1:500 in PBST and added to each well. After incubating and washing, 100 µl of substrate of ortho-phenylenediamine (OPD) (Sigma-Aldrich, USA) was added to wells. The catalytic enzyme was stopped by adding 50 µl of 20% sulfuric acid at a specific time and the absorbance was measured at 490 nm using automated ELISA reader (BIOTEC LX800, USA). Furthermore, all samples were approved for the determination of anti-*Toxoplasma* IgG using commercial kits (Trinity Biotech Captia, New York, USA) according to the manufacturer’s instructions.

#### Testosterone assessment

Concentration of testosterone was assessed at 37 °C using Roche Cobas^®^ e 411 Immunoassay (Roche Diagnostics, Mannheim, Germany) according to the manufacturer’s instructions. In the first incubation step, 20 μl of the sample were incubated with a biotinylated monoclonal testosterone-specific antibody and 2-bromoestradiol (to release testosterone). In the second step, streptavidin-coated microparticles and a ruthenylated testosterone derivative were added to the mixture. The reaction mixture was transferred to a measuring cell and the microparticles were magnetically captured on the surface of an electrode. Chemiluminescence was measured using photomultiplier and the concentration of testosterone was calculated using calibration curve [22]. Interpretation of the testosterone concentration was based on the manufacturer’s recommendation as follows: normal range for men, 2.49–8.36 ng/ml; and for women, 0.084–0.481 ng/ml. Experiments were carried out in triplicate, and the mean was calculated for each sample.

### Statistical analysis

Statistical analyses were carried out using SPSS Software v.16. Data were analyzed using multiple univariate analyses of variance (ANOVA) and Chi square test. Pearson product-moment correlations were used between optical density of ELISA and concentration of testosterone. Comparison of quantitative variants between two groups was assessed by student *t* test. Data description was carried out by calculating frequencies and 95% confidence intervals. Differences were considered as significant when P ≤ 0.05.

## Results

Results of anti-*Toxoplasma* IgG antibody detection and serum testosterone concentration in infected and non-infected subjects are shown in Table 1. Differences in mean concentrations of testosterone were reported between infected and non-infected subjects (P < 0.05) as testosterone concentration was significantly higher in IgG-positive group than that in IgG-negative one. In infected subjects, 13.2 and 26.3% of men and women had high concentrations of serum testosterone, respectively. The mean concentration of serum testosterone was higher in men and women infected by *T. gondii* and statistically significant (P = 0.02 and P = 0.04, respectively), compared to that in control group. Furthermore, correlation between the mean OD of ELISA and concentration of testosterone was significant in infected men and women with values of 0.007 and 0.004, respectively. No statistically significant association was found between IgG titers and testosterone levels in men and women in *Toxoplasma* seropositivity group in comparison with control group (P > 0.05).

**Table 1:** Mean OD of ELISA and concentration of testosterone (ng/ml) in 18–49 year-old infected men and women, compared to that in non-infected ones

Group	Mean OD ± SD	Mean concentration of testosterone (ng/ml) ± SD
Infected men	1.05 ± 0.53	5.6 ± 1.99
Infected women	0.94 ± 0.37	0.41 ± 0.22
Non-infected men (sero-negative)	0.14 ± 0.08	4.56 ± 1.96
Non-infected women (sero-negative)	0.15 ± 0.08	0.31 ± 0.17

## Discussion

Parasite-induced changes to the host endocrine system provide a possible mechanism of altering host behaviors. Significant sex differences have been reported regarding host changes in response to *T. gondii* infection [19]. Testosterone is an important influencing factor in behavior and personality in both sexes. As shown in majority of the studies, increased testosterone was associated with antisocial, aggression and dominance behaviors [23–25]. Altered testosterone levels have been observed in *T. gondii* infections; however the literatures lack consensuses. Evidence suggests that testosterone activation may cause sexual arousal directed towards feline odor in some rodents [26]. Interestingly, castrated male rats do not exhibit loss of fear phenotype, suggesting that testosterone plays a direct role in this behavior [27]. Results of the current study have shown that mean concentrations of serum testosterone are significantly higher in men and women infected by toxoplasmosis, compared to that in control group. Increased concentration of testosterone during latent toxoplasmosis can result in inducted behavioral alterations and immunosuppressive effects characterized by lower cellular immunity [25, 28]. Administration of exogenous testosterone can reduce fear in humans and rodents [29]. It is possible that men with increased levels of testosterone have greater chance of *Toxoplasma* infection either due to impaired immunity or changed behavior and personality profile. For instance, personal tendency to disregard rules of the society can result in lower hygienic standards and hence increase risk of contact with sources of infection [30–33]. The underlying mechanism for these behavioral alterations are usually thought to be variations in neurotransmitter functions and more specifically due to high levels of dopamine. In addition, there are indications that enhanced testosterone levels play an important role in behavioral abnormalities [34].

Several studies have shown that, direct and indirect evidence exist on increased testosterone in *Toxoplasma* infected human and animals [35–39]. *T. gondii* produces high concentrations of testosterone in infected hosts and enhanced mRNA expression of luteinizing hormone receptor (LHR), which regulate the synthesis of testosterone in testes on Leydig cells [27]. The *Toxoplasma* infected men are about 3 cm taller than *Toxoplasma* free men and having further muscles and dominant faces [38, 39]. *Toxoplasma* infected men and women have a lower second to fourth digit length ratio in the left hand (2D:4D ratio) and are more likely to give birth to boys than girls [14, 40]. The findings of current study is in accordance with James hypothesis (2010) that many parasites and pathogens could change the concentration of steroid hormone. He has demonstrated that infected hosts, often with shifted sex ratio, increase number of males in generations [40]. Testosterone is a hormone which is responsible for the growth of secondary male sexual characteristics. An alternative hypothesis explaining *Toxoplasma* associated sex ratio shift suggests that the phenomenon is caused by the higher possibility of survival of more immunogenic male embryos by inducing immunosuppression mechanisms [30]. Indeed, both hypotheses may be compatible since the proximate mechanism of immunosuppression remains unknown and may involve the parasite-induced shift in steroid hormones.

The results of present study do not agree with Flegr et al. showed that *Toxoplasma* infected men had a higher concentration of testosterone while women had a lower concentration of the hormone, compared to control group [19]. Furthermore, Flegr suggested that the personality profiles of infected men and women are different and the opposite direction of the testosterone shift in men compared to women can explain the observed gender specificity of behavioral changes in people infected with *Toxoplasma* parasite. They have concluded that infected women are warm-hearted, conscientious, outgoing, persistent, and moralistic while infected men are more likely to disregard rules and were more expedient, suspicious, jealous, and dogmatic [19].

Contrary to our results, a controversial study by Kankova et al. has shown a decrease testosterone levels (total testosterone and free testosterone) in female and male laboratory mice infected by virulent strains of *Toxoplasma* at a latent phase, compared to uninfected controls. This controversy may be seen due to the different parasite strain, which differs in virulence and epidemiological occurrence [41]. Therefore, the parasite genotype seems to be an important parameter influencing the clinical infection in humans [42]. It is possible that the physiological reaction to *Toxoplasma* infection qualitatively differs between mice and humans. Other reports show that reduced serum and testicular testosterone levels was found in male rats infected by high doses of *T. gondii*, RH strain compared to controls [43]. Similarly, Oktenli et al. have demonstrated that concentration of follicle stimulating hormone (FSH), luteinizing hormone (LH), free testosterone (FT) and total testosterone (TT) were significantly lower than controls in serum of male patients during acute toxoplasmosis [44]. Results of the current study showed that the mean concentration of serum testosterone was higher in men and women infected by toxoplasmosis, compared to that in control group. Of various mechanisms described in *T. gondii* for behavioral alterations, increased testosterone seems to play a significant role. Alterations of testosterone during latent toxoplasmosis can affect several behavioral, physiologic and immunological parameters in a long time.

## Limitations

In this study the association of latent toxoplasmosis and psychological disorders was not tested in patients. It is suggested to further investigation of direct correlation between latent toxoplasmosis and psychological disorders in animal and human models.

## Abbreviations

ECLIA: electro chemiluminescence immunoassay
ELISA: enzyme-linked immunosorbent assay
IgG: immunoglobulin G
PBST: phosphate buffered saline, tween 20
HRP: hourseradish peroxidase
OPD: ortho phenylenediamine
OD: optical density
2D:4D ratio: second to fourth digit length ratio
SPSS: statistical package for the social sciences
FSH: follicle stimulating hormone
LH: luteinizing hormone
